# Study of Occupational Chromium, Iron, and Nickel Exposure and Amyotrophic Lateral Sclerosis in Denmark

**DOI:** 10.3390/ijerph17218086

**Published:** 2020-11-02

**Authors:** Aisha S. Dickerson, Johnni Hansen, Ole Gredal, Marc G. Weisskopf

**Affiliations:** 1 Department of Epidemiology, Johns Hopkins Bloomberg School of Public Health, Baltimore, MD 21205, USA; 2 Danish Cancer Society Research Center, 2100 Copenhagen, Denmark; johnni@cancer.dk; 3 National Rehabilitation Center for Neuromuscular Disorders, 8000 Copenhagen, Denmark; olgr@rcfm.dk; 4 Departments Epidemiology of Environmental Health, Harvard T.H. Chan School of Public Health, Boston, MA 02115, USA; mweissko@hsph.harvard.edu

**Keywords:** amyotrophic lateral sclerosis, ALS, motor neuron disease, metals, occupational exposures

## Abstract

Studies of occupational metal exposures and amyotrophic lateral sclerosis (ALS) have focused primarily on known neurotoxicants, including lead, mercury, selenium, and cadmium. However, these exposures are often co-occurring with other lesser studied metals. We conducted a population-based case-control study with the aim of assessing associations between occupational chromium, iron, and nickel exposures and risk of ALS. We identified ALS cases in Denmark from 1982 through 2013 from the Danish National Patient Registry and matched them to 100 controls based on birth year and sex. Cumulative metal exposures were estimated using job exposure matrices applied to occupational history from the Danish Pension Fund. Although mutually adjusted odds of ALS were higher in men with chromium exposures in the third quartile (aOR = 1.24; 95% CI 0.91, 1.69) and fourth quartile (aOR = 1.19; 95% CI: 0.80, 1.76) compared to those with no exposure, differences did not reach statistical significance. We also observed higher odds of ALS in women with nickel exposures in the third quartile (aOR = 2.21; 95% CI: 1.14, 4.28), but not for the fourth quartile (aOR = 0.61; 95% CI: 0.23, 1.64). Our findings do not suggest associations between occupational exposures to these metals and ALS. However, unavoidable non-differential misclassification from the use of JEMs may have masked truly increased risk.

## 1. Introduction

Amyotrophic lateral sclerosis (ALS) is a rare degenerative motor neuron disease with notable progressive muscle weakness and decline in motor function [1]. ALS typically results in rapid deterioration to death 3 to 5 years after onset [2]. Although 5 to 10% of ALS cases are attributed to familial genetic susceptibility, little is understood about the exogenous factors that influence risk of ALS [3]. However, it has been suggested that a large portion of these sporadic ALS cases may be associated with occupational and environmental exposures [1,4,5].

Occupational exposure to metals, including chromium, iron, and nickel, is often co-occurring in industries involving welding, machinery, pigments, and metal product manufacturing [6]. Hexavalent chromium [Cr(VI)], chromium(III), chromium compounds, and nickel compounds are well-known carcinogens [7], with demonstrated mechanistic impacts on oxidative stress [8,9]. Additionally, excessive iron exposures can impair cellular metabolism and increase subsequent cellular aging and death [10]. Although iron is an essential nutrient for normal neuronal function, studies have suggested iron dyshomeostasis and accumulation in the brain can eventually lead to neurodegeneration [11,12]. Furthermore, though each of these toxicants can increase DNA damage and induce cellular death [13], they have been shown to interact synergistically to adversely impact cytotoxicity [14].

Previous studies have indicated increased ALS risk in people with jobs commonly exposed to metals, including those in welding [15], construction [4], manufacturing [16,17,18], and the military [19,20]. Several other studies have also suggested that occupational metal exposures may play a role in ALS [16,17,21,22,23,24], but many of these only evaluated well-known neurotoxicants like lead [25,26,27], mercury [27], and selenium [28]. Additionally, no previous study of metal exposures and ALS has used job exposure matrices (JEMs) to objectively estimate exposure to a variety of common occupationally encountered metals based on the entire work history. In our population-based study using Danish registry data, we utilize JEMs to estimate cumulative occupational exposure to lesser studied metals in relation to ALS—chromium, nickel, and iron—with the aim of assessing their association with risk of ALS both alone and in combination.

## 2. Methods

### 2.1. Study Participants

We used the Danish National Patient Registry to identify ALS diagnoses in Denmark. The registry started in 1977, but we only included cases first identified in 1982 or later in order to avoid prevalent cases at the start of the registry, and included cases identified through 2013. Identification was via the extended Danish version of *International Classification of Diseases and Related Health Problems, Eighth Revision* (ICD-8) code of 348.0 (amyotrophic lateral sclerosis) from 1977 to 1994 and *Tenth Revision* (ICD-10) code of G12.2 (motor neuron disease) from 1994 through 2013 [29]. The first recorded date of ALS diagnosis was defined as the “index date,” and we matched each ALS case to 100 controls free of ALS and alive at the index date, randomly selected from the Danish Central Person Registry matched on birth year and sex [30]. To reduce potential exposure misclassification from jobs held before the start of the Danish Pension Fund, which started in 1964 [31], we excluded 280,073 subjects who were ≥25 years of age in 1964. We also removed 187 cases and 14,406 controls with <5 years of work experience from our analysis to diminish healthy-worker hire bias. This study was approved by the Danish Data Protection Agency and conducted using secondary analysis of pre-existing data, and thus was determined to be exempt by the Harvard T.H. Chan School of Public Health Institutional Review Board.

### 2.2. Exposure Assessment

We obtained employment history, including type of industry and duration of employment within each industry, from the Danish Pension Fund [31] and calculated cumulative occupational exposure to chromium, nickel, and iron using JEMs. These JEMs were originally developed for the Nordic Occupational Cancer Study, then revised for Denmark based on occupational biomonitoring, industrial measurements, and expert evaluation [32,33]. Time-specific exposure level and probability estimates are divided at 1945, 1960, 1975, 1985, and 1995 [32]. For each identified chromium, nickel, and iron-exposed industry, a probability and estimated level of exposure is assigned for each metal at designated timepoints. For example, someone employed in metal smelting between 1985 and 1994 would have an estimated chromium exposure probability of 0.38 and daily exposure level of 40 ppm. For each of these metals, common exposed industries include metal smiths, molders, toolmakers, plumbers, sheet metal workers, machine assemblers, and metal plating and coating workers.

We first considered a binary ever/never exposure to a metal as anyone who had ever held an occupation with any probability of exposure to each metal. Because people may change jobs as a result of exposures, which can lead to healthy worker survivor bias [34,35], and due to the potential impact of differing windows of exposure on disease onset, we considered exposure lags of 0, 5 and 10 years. For example, with a 5-year exposure lag, occupations with any probability of exposure to a given metal were not counted towards exposure if they were held within 5 years of the index date. We then estimated continuous exposures to each metal for each subject by multiplying the time-specific probability by the level of exposure for each occupation provided in the Danish Pension Fund record [32]. To calculate cumulative exposure, we further multiplied estimated exposure by the duration of employment in that industry, then summed all estimated exposures through the index date for each study subject. Because we saw little difference with the different lags in analyses of ever/never exposures, we only considered the 5-year lag for the analysis of cumulative exposures.

### 2.3. Statistical Analysis

We stratified all analyses by sex to account for expected differences in jobs and job tasks between men and women. To assess risk of ALS associated with exposure to chromium, iron, and nickel, we obtained odds ratios (OR) and 95% confidence intervals (95% CI) using unconditional logistic regression. We also categorized 5-year lagged cumulative metal exposures into quartiles of those with any exposure and evaluated ALS risk of those with third and fourth quartile exposure levels using no exposure as the reference group. Cut points for quartiles were based on sex-specific distributions. Each metal was initially analyzed individually with adjusted for sociodemographic confounders, including age at the index date, geographic residence at the time of birth (Copenhagen, Copenhagen suburbs, Aarhus/Odense, provincial towns, rural areas, and Greenland), and socioeconomic status based on the higher of the subject’s or spouse’s occupation title (e.g., academics and corporate managers, high-salary positions, low-salary positions, skilled workers, and unskilled workers) at the index date. Then metals were mutually-adjusted by including each metal simultaneously in the model along with previously mentioned confounder. All statistical analyses were conducted with a 5% level of significance using SAS 9.4 statistical software (SAS Institute Inc., Cary, NC, USA).

## 3. Results

After implementing our exclusion criteria, we were left with 1639 ALS cases and 168,194 controls. Table 1 shows the demographic distribution of study subjects by sex. Per our sampling strategy, the portion of ALS cases included in our analysis was close to 1%. Age distributions between men and women did not differ greatly. A large portion of both men and women were classified into households with the highest occupational level being “skilled worker” at 31%. Approximately 40% of subjects were born in provincial towns, and about 24% lived in Copenhagen suburbs. Although the majority of men and women were married at the index date (68% and 66%, respectively), a larger portion of men were never married (16% vs. 10%, respectively).

Metal-specific results for our analysis of any exposure to chromium, iron, and nickel any time prior, up to 5 years prior, and up to 10 years prior to the index date are displayed in Table 2. As expected, men were more likely than women to be occupationally exposed to chromium (48% vs. 10%), iron (27% vs. 7%), and nickel (29% vs. 9%). There were no notable exposure-risk patterns observed for men exposed to chromium, iron, or nickel, individually or mutually adjusted. Likewise, there were no observed associations for individual occupational metal exposures in women. However, when mutually adjusted, our analysis revealed higher, though not precise, adjusted odds of ALS for women with exposure to nickel (5-year lag adjusted OR (aOR) = 1.29; 95% confidence interval (CI) 0.75–2.19).

As shown in Table 3, when examining 5-year lagged cumulative estimated exposures of chromium, iron, and nickel, odds ratios were generally elevated for higher quartile levels of exposure to chromium (third quartile aOR = 1.24; 95% CI: 0.91, 1.69 and fourth quartile aOR = 1.19; 95% CI: 0.80, 1.76) compared to those with no exposure. For women, we observed higher odds of ALS in those with cumulative nickel exposures in the third quartile (aOR = 2.21; 95% CI: 1.14, 4.28); however, with an inverse and non-significant result (aOR = 0.61; 95% CI: 0.23, 1.64), results for the fourth quartile were not consistent. All other quartile exposures exhibited higher, but not significant aORs, indicating no trend in this association. There were no other notable patterns seen for other metal exposures and ALS in men or women.

## 4. Discussion

In this prospective study of occupational metal exposures and ALS risk in a Danish population, results suggest that men with the highest levels of exposure to chromium may be at modestly greater risk for ALS. In women, there was a significant positive association seen for third quartile exposures to nickel, which may be due to chance. However, as these are occupational exposure estimates, it is possible that the highest quartiles not having the highest odds could relate to healthy worker survivor bias [34,35], in which those less susceptible to the outcome are preferentially the ones with most exposure because of job switching [36]. The direction and magnitude of effect was similar for results from all analyses where the exposures were lagged to exclude exposures within certain windows prior to the index date (no lag, 5-year lag, and 10-year lag). To the extent that the results reflect some associations, it is important to note that the JEMs used to estimate occupational metal exposures in this study are not sex specific. Thus, any observed sex differences in our study may well be due to differences in job tasks and industries of employment between men and women in Denmark rather than biological differences.

People in occupations involving welding and metal working are often exposed to chromium, iron, and nickel concurrently through inhalation of welding fumes [6]; however, few studies have investigated ALS in relation to specific exposures to chromium, iron, or nickel. Although some previous studies have indicated increased risk of ALS in welders [37,38,39,40] and machine assemblers [15], while others showed no association [16,17,41], these have focused primarily on exposures to electric shocks and magnetic fields. Conversely, risk seen in these occupations could potentially be attributed to metal exposures, which have previously been suggested as oxidative, inflammatory, and neurodegenerative [42,43]. Specifically, iron, a biologically essential metal, can modulate cellular respiration at high levels and increase cellular damage which may lead to neuronal death and has previously been linked to other neurodegenerative disorders like Parkinson’s disease [42,44] and Alzheimer disease [11]. Chromium, a well-known carcinogen, has been demonstrated in mouse models to induce brain injury through oxidative stress and inflammation [45]. Furthermore, nickel, an essential yet potentially carcinogenic metal, can accumulate in neuronal tissue, cause cellular death, and inhibit neurotransmission [46]. Additionally, nickel is a recognized allergen and irritant [47], and some have suggested that inflammation and immune responses may play a role in ALS pathology [48]. Collectively, these factors suggest that these particular metals warrant investigation in relation to ALS.

While some previous investigations of overall metal exposures and risk of ALS reported null results [23,49,50], others indicated higher risk [16,17,26,51,52]. However, of the three metals we investigated in our study—chromium, iron, and nickel—only one previous study reported individual results for self-reported occupational exposure to chromium in Washington State and found more than two times increased risk of ALS [53]. Ours is the first study, to our knowledge, to report results on an investigation of occupational exposures to chromium and iron in relation to ALS risk, and we used prospectively collected occupational history rather than self-reported exposure. Although odds of ALS were higher in men with higher quartile exposures to chromium, these results were not statistically significant and increased risk seen in women exposed to higher levels of nickel were not consistent. Thus, our results are not supportive of a strong association between occupational exposure to these metals and ALS.

Although our study presents results of previously unexplored exposure-disease relationships using prospective population-based data to assess cumulative exposures, our study is not without limitations. We used JEMs to estimate occupational metal exposures, which certainly has some exposure measurement error; in particular, being unable to differentiate between exposure difference within the same type of jobs. However, all JEMs created for use in the Nordic Occupational Cancer Study are mostly based on measurements over many decades evaluated by exposure experts [32]. Some exposure misclassification may also be present due to our inability to assess exposures in jobs held prior to the 1964 establishment of the Danish Pension Fund. However, we minimized this potential bias by only including cases and controls who would have been at least 25 years of age when Pension Fund registration began. We would expect that any residual missed occupational history would not have differed between ALS cases and controls, which thus would, if anything, have biased any true association towards the null. Lastly, these JEMs were based on using average non-sex-specific estimates of exposures based on industry-level measurements and did not account for variation in personal use of protective equipment. This may further have led to some misclassification of individual exposures, which also may have weakened our effect estimates. However, we believe the strengths of this study to greatly outweigh the limitations mentioned.

We reported the first analysis of ALS and prospective occupational exposures to chromium, iron, and nickel using population-based data from Denmark. Although there was some suggestion that there may be increased risk of ALS in men exposed to chromium and women exposed to nickel, results were not supportive of a strong association. Thus, these results should be interpreted with caution. Given our limitations in assessing specific job tasks within each industry as well as use of personal protective equipment, future studies should collect this data to better estimate individual-level exposures.

## Figures and Tables

**Table 1:** Demographic Characteristics at the Index Date by Sex.

Characteristic	Men (n = 101,678)		Women (*n* = 68,155)	
	*n*	%	*n*	%
Age (years)				
<45	15,109	14.9%	7705	11.3%
45–54	26,592	25.2%	16,614	24.4%
55–64	39,186	38.5%	27,878	40.9%
65–74	20,791	20.4%	15,958	23.4%
Socioeconomic status *				
Academics and managers	11,154	11.0%	8069	11.8%
High-salary positions	14,366	14.1%	10,465	15.4%
Low-salary positions	17,092	16.8%	13,282	19.5%
Skilled workers	31,625	31.1%	20,899	30.7%
Unskilled workers	16,094	15.8%	10,244	15.0%
Unknown	11,347	11.6%	5196	7.6%
Residence at birth				
Copenhagen	10,148	10.0%	6839	9.4%
Copenhagen suburbs	24,002	23.6%	16,907	24.8%
Aarhus/Odense	10,040	9.9%	6725	9.9%
Provincial towns	41,607	40.9%	27,518	40.4%
Rural areas	15,443	15.2%	10,016	14.7%
Greenland	132	0.13%	23	0.03%
Unknown	306	0.3%	127	0.2%
Marital status				
Married	69,087	68.0%	44,874	65.9%
Unmarried	16,396	16.1%	6768	9.9%
Divorced	13,349	13.1%	10,886	16.0%
Widowed	2654	2.6%	5542	8.1%
Unknown	192	0.2%	85	0.1%

* Socioeconomic designation is for the highest level of either the subject or the subject’s spouse.

**Table 2:** Odds of ALS based on logistic regression, Denmark, 1982–2013.

	Men			Women		
Metal	Occupationally Exposed to the Metal (yes/no)	aOR (95% CI)	Mutually Adjusted aOR (95% CI)	Occupationally Exposed to the Metal (yes/no)	aOR (95% CI)	Mutually Adjusted aOR (95% CI)
**No Lag**						
Chromium	34,425/67,253	1.06 (0.93, 1.21)	1.01 (0.79, 1.30)	7089/61,066	1.01 (0.70, 1.18)	0.93 (0.59, 1.46)
Iron	27,087/74,591	1.07 (0.93, 1.24)	1.11 (0.68, 1.82)	4884/63,271	0.91 (0.66, 1.24)	0.79 (0.37, 1.65)
Nickel	29,489/72,189	1.06 (0.92, 1.22)	0.95 (0.62, 1.46)	6076/62,079	0.97 (0.74, 1.28)	1.24 (0.73, 2.12)
**5-Year Lag**						
Chromium	33,342/68,336	1.07 (0.93, 1.23)	1.02 (0.79, 1.31)	6785/61,370	0.91 (0.70, 1.19)	0.88 (0.55, 1.41)
Iron	26,221/75,457	1.08 (0.94, 1.25)	1.14 (0.70, 1.88)	4680/63,475	1.00 (0.69, 1.44)	0.82 (0.39, 1.76)
Nickel	28,558/73,120	1.07 (0.93, 1.23)	0.93 (0.60, 1.45)	5835/62,320	1.00 (0.76, 1.32)	1.29 (0.75, 2.19)
**10-Year Lag**						
Chromium	31,608/70,070	1.06 (0.92, 1.21)	0.95 (0.73, 1.25)	6299/61,856	0.97 (0.74, 1.27)	0.97 (0.60, 1.56)
Iron	24,875/76,803	1.09 (0.94, 1.26)	1.23 (0.73, 2.05)	4384/63,771	0.97 (0.70, 1.33)	0.81 (0.37, 1.75)
Nickel	27,124/74,554	1.07 (0.93, 1.24)	0.92 (0.59, 1.44)	5494/62,661	1.03 (0.77, 1.36)	1.24 (0.71, 2.16)
**>50% Probability of Exposure**						
**No lag**						
Chromium	5293/96,385	0.93 (0.69, 1.26)	0.87 (0.63, 1.20)	574/67,581	0.53 (0.17, 1.66)	0.59 (0.18, 1.96)
Iron	23,994/77,684	1.07 (0.92, 1.24)	1.10 (0.94, 1.29)	3816/64,339	0.84 (0.58, 1.20)	0.89 (0.61, 1.30)
**5-Year lag**						
Chromium	4856/96,822	0.95 (0.70, 1.29)	1.12 (0.95, 1.31)	521/67,634	0.59 (0.19, 1.84)	0.92 (0.63, 1.35)
Iron	23,222/78,456	1.09 (0.94, 1.26)	0.87 (0.62, 1.21)	3656/64,499	0.87 (0.61, 1.26)	0.63 (0.19, 2.09)
**10-Year lag**						
Chromium	4175/97,503	0.98 (0.71, 1.36)	1.11 (0.94, 1.30)	446/67,709	0.69 (0.22, 2.14)	0.94 (0.64, 1.38)
Iron	22,041/79,637	1.09 (0.93, 1.26)	0.90 (0.64, 1.28)	3433/64,722	0.90 (0.62, 1.31)	0.73 (0.22, 2.42)

All models are adjusted for age, residence at birth, and socioeconomic status.

**Table 3:** Odds of ALS by 5-year lagged quartile exposures, Denmark, 1982–2013.

		Individually		Mutually Adjusted	
	Occupationally Exposed to the Metal (yes/no)	Third Quartile aOR (95% CI)	Fourth Quartile aOR (95% CI)	Third Quartile aOR (95% CI)	Fourth Quartile aOR (95% CI)
**Men**					
Chromium	33,342/68,336	1.24 (1.00, 1.54)	1.01 (0.79, 1.28)	1.24 (0.91, 1.69)	1.19 (0.80, 1.76)
Iron	26,221/75,457	1.21 (0.88, 1.44)	0.92 (0.69, 1.21)	0.96 (0.54, 1.73)	0.89 (0.47, 1.69)
Nickel	28,558/73,120	1.22 (0.97, 1.55)	0.91 (0.70, 1.19)	1.05 (0.62, 1.78)	0.82 (0.45, 1.48)
**Women**					
Chromium	6785/61,370	0.91 (0.54, 1.52)	0.78 (0.45, 1.35)	0.61 (0.29, 1.26)	0.56 (0.21, 1.27)
Iron	4680/63,475	1.24 (0.73, 2.12)	1.05 (0.59, 1.87)	1.05 (0.40, 2.74)	1.85 (0.63, 5.44)
Nickel	5835/62,320	1.73 (1.14, 2.61) *	0.56 (0.28, 1.14)	2.21 (1.14, 4.28) *	0.61 (0.23, 1.64)

All models are adjusted for age, residence at birth, and socioeconomic status. * *p* < 0.05.
